# Cutaneous Chronic Graft Versus Host Disease in a Symmetric Distribution

**DOI:** 10.7759/cureus.4614

**Published:** 2019-05-07

**Authors:** Nehal R Shah, Alexa Leone, Robert Rothbaum, Gregory RR. Delost, Kevin Cooper

**Affiliations:** 1 Medicine, Touro College of Osteopathic Medicine, New York, USA; 2 Dermatology, University Hospitals Cleveland Medical Center, Cleveland, USA

**Keywords:** scleroderma, symmetrically distributed, chronic graft-versus-host disease, morphea, generalized morphea, graft versus host disease, sclerodermoid

## Abstract

Graft-versus-host disease (GVHD) is a common complication following patients who have undergone allogenic hematopoietic stem cell transplantation (allo-HSCT). While GVHD has been previously sub-categorized through a temporal relationship upon transplantation, revisions from the National Institutes of Health have modified the diagnosis criteria to be more involved with specific signs and symptoms. Chronic classifications of GVHD include non-sclerotic and sclerotic forms, and the sclerotic form can be further classified based on morphologies such as lichen-sclerosis-like, sclerodermoid or morphea-like plaques. Generalized morphea can have similar histopathological findings but in order to be diagnosed, certain diagnostic criteria must be met. Herein, we report a patient with linear and inflammatory morphea morphology of chronic GVHD, which presents symmetrically on both lower extremities.

## Introduction

Graft-versus-host disease (GVHD) is a common complication of patients undergoing an allogenic hematopoietic stem cell transplantation (allo-HSCT), with a 40% to 60% prevalence and a major cause of morbidity and mortality [1]. GVHD has been sub-categorized as acute GVHD (aGVHD) or chronic GVHD (cGVDH), with aGVHD occurring prior to 100 days of the allo-HSCT, while cGVHD occurring after 100 days [2]. Revisions from the National Institutes of Health (NIH) proposed that aGVHD and cGVHD should be differentiated based on the clinical features and histopathology rather than the temporal relationship with the transplantation. To diagnose cGVHD requires at least one “diagnostic” (established signs and symptoms unique to cGVHD) manifestation, one “distinctive” (not ordinarily found in aGVHD but insufficient to equivocally establish a cGVHD diagnosis) manifestation, plus a pertinent skin biopsy, laboratory or other test, and evaluation by a specialist or radiographic imaging evidence [3].

Current classification for cGVHD includes non-sclerotic and sclerotic forms. The non-sclerotic forms may precede the onset of the sclerotic form. Sclerotic cGVHD can be further classified into multiple morphologies which include lichen sclerosis-like, and sclerodermoid or morphea-like plaques [4]. Generally, the morpheaform variant does not present symmetrically, linearly, or on distal extremities. Here we report a patient with linear and inflammatory morphea morphology, which presents symmetrically on both lower extremities.

## Case presentation

**Figure 1:**
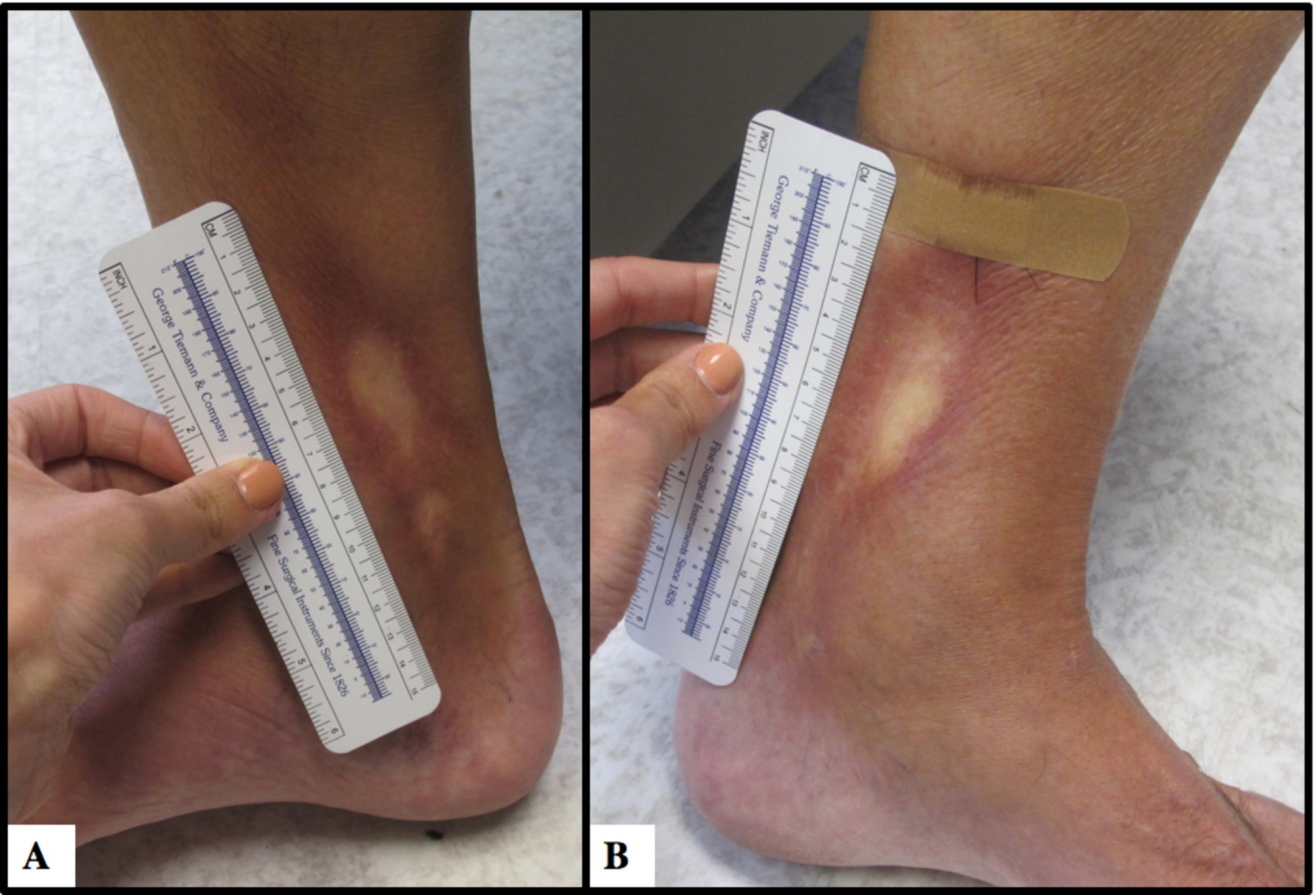
Symmetrically distributed cGVHD A: Right medial ankle, well-demarcated, bound-down, patches with central hypopigmentation and peripheral erythema; B: left medial ankle symmetrical lesion cGVHD, chronic graft-versus-host disease

A 58-year-old Caucasian male presented to dermatology complaining of rash and limited mobility of his bilateral ankles of nine months duration. He had a history of acute myeloid leukaemia, status post reduced intensity conditioning matched unrelated donor hematopoietic stem cell transplant (allo-HP SCT) with complete remission, as well as a history of low-grade papillary urothelial invasive bladder carcinoma and multiple sclerosis treated with ocrelizumab for 18 months. Of note, allo-HP SCT (2011) was complicated by grade II aGVHD and mild extensive cGVHD with Sicca syndrome affecting the oral cavity, lacrimal glands, joints and scalp hair, with a 2014 conjunctival biopsy showing chronic conjunctivitis with prominent lymphocytic follicles and scar. On examination, involving the bilateral medial ankles, were linear, well-demarcated, bound-down, patches with central hypopigmentation and peripheral erythema (Figures 1A-1B). The skin appeared taught; on a range of motion examination, there was limited motion with flexion, while extension remained intact. There were no oral or ocular lesions and no nail changes.

**Figure 2:**
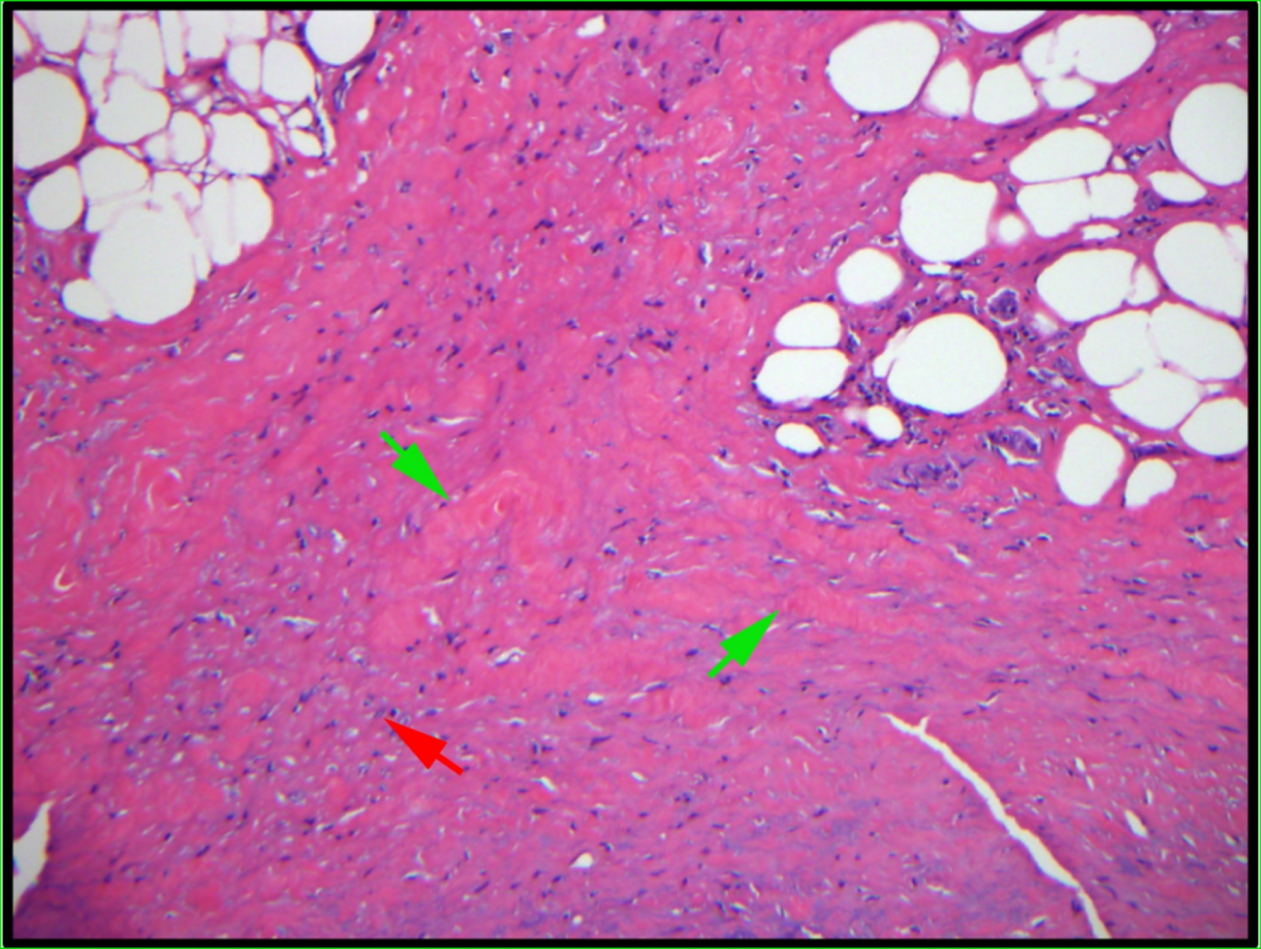
Left ankle punch biopsy A punch biopsy on the left ankle revealed prominent septal fibrosis (green arrows) and fascial fibrosis with mild chronic inflammation (red arrow; 20X).

A punch biopsy on the left ankle revealed prominent septal and fascial fibrosis with mild chronic inflammation (Figure 2). Laboratory testing including complete blood count with differential, complete metabolic panel, and lactate dehydrogenase were within normal limits.

We recommended starting tacrolimus 0.1% ointment and tretinoin 0.1% cream twice daily to the affected areas of the ankles. The patient was also continued on an oral prednisone taper, which had been initiated by another provider. In addition, the patient was referred to physical therapy to help measure baseline movement and restriction along with providing exercises to help increase range of motion.

## Discussion

Although the kinetics between aGVHD and cGVHD have overlapping entities, it is unclear whether they are sequential phases of the same disease or independent disorders with unique molecular and pathophysiological mechanisms. Simplistically, host antigen-presenting cells prime the proliferation and migration of alloantigen-specific donor T-cells to site-directed immune-mediated injury [5]. The prevalence of cutaneous manifestations is as high as 90% to 100% for patients diagnosed with cGVHD. The clinical distribution for cutaneous cGVHD can vary based on the variant. The Sclerodermoid disease variant manifests as localized or generalized morphea-like plaques that tend to affect the trunk and proximal extremities or can be limited to areas of previous inflammation, infection or trauma [4]. While our patient denied previous inflammation, infection or trauma in his ankles, his previous medical history with allo-HP SCT in 2011 along with both his aGVHD and mild cGVHD with sicca syndrome diagnoses indicated that our patient was likely experiencing further manifestations of cGVHD. 

Other differential diagnoses with similar cutaneous and histological manifestations of cutaneous cGVHD include scleroderma. Scleroderma is an autoimmune disorder classified into two categories: systemic scleroderma and localized scleroderma. Localized scleroderma usually consists of lesions limited to the skin, subcutaneous tissue, and sometimes bone beneath the lesion. It is further classified into three categories: morphea, linear scleroderma and generalized morphea. To meet the qualifications of generalized morphea, patients must fulfil two requirements: (i) four or more lesions more than 3 cm in diameter; and (ii) involvement of two out of seven anatomical sites. Patients who do not meet these two criteria are categorized into either morphea or linear morphea [6-7]. Unfortunately, the criteria for morphea subtype are not well defined [7]. Linear morphea is typically seen in children and adults [8]. It is generally unilateral in 95% of the cases and consists of a deep linear streak that can extend through the dermis, subcutaneous tissue, underlying muscle and bone, contrary to our patient who exhibited symmetrical display of leg lesions [8,9].

Our patient did not meet the criteria for generalized morphea since he only displayed two lesions. A previous study performed a retrospective analysis of 110 patients diagnosed with cGVHD involving any organ system. Of 81 patients had evidence of cutaneous findings, 58 displayed cutaneous sclerosis-like features. Eleven (19%) of the 58 patients were further categorized into having morphea-like lesions. These cases demonstrated distribution either on the lower abdomen (“waistband” area) or the lateral torso (“brassiere-band”) regions and often in a linear pattern [10]. The symmetrically distributed leg lesions with prominent morpheaform morphology seem to be unique in this patient and represent an atypical presentation of chronic sclerodermatous GVHD.

## Conclusions

Cutaneous manifestations of chronic graft-versus-host disease can mimic numerous other modalities. While the criteria for establishing chronic graft-versus-host disease are stringent, it is important to perform a full workup to establish the correct diagnosis.

## Appendices

Abbreviations and acronyms:

GVHD: Graft-versus-host disease

Allo-HP SCT: Allogenic hematopoietic stem cell transplantation

NIH: National Institutes of Health

aGVHD: Acute graft-versus-host disease

cGVHD: Chronic graft-versus-host disease
